# *Pneumocystis jirovecii* in a patient on dose‐dense chemotherapy for early breast cancer

**DOI:** 10.1002/rcr2.459

**Published:** 2019-07-05

**Authors:** Chloe Khoo, Jenny Gilchrist, Jonathan Philip Williamson, Miriam Paul, Richard Kefford

**Affiliations:** ^1^ Cancer Program, MQ Health Macquarie University Hospital Sydney New South Wales Australia; ^2^ MQ Respiratory and Sleep Macquarie University Hospital Sydney New South Wales Australia; ^3^ Macquarie University Hospital Sydney New South Wales Australia

**Keywords:** Adjuvant chemotherapy, breast cancer, corticosteroids, dose‐dense chemotherapy, *Pneumocystis jirovecii*

## Abstract

A 70‐year‐old woman underwent adjuvant chemotherapy with dose‐dense doxorubicin and cyclophosphamide for early breast cancer. After her fourth cycle of chemotherapy, she developed severe fatigue and cough with rapid‐onset hypoxic respiratory failure. Investigations demonstrated extensive bilateral consolidation with positive bronchial washings for *Pneumocystis jirovecii* by polymerase chain reaction (PCR). Despite high‐dose trimethoprim‐sulfamethoxazole, she progressed to multi‐organ failure and succumbed. *Pneumocystis jirovecii* pneumonia (PJP) has traditionally rarely occurred in women on adjuvant breast cancer chemotherapy but may pose a more serious risk in dose‐dense regimes due to higher concurrent exposure to anti‐emetic corticosteroids. Clinicians are alerted to the need for vigilance of this rare complication and for rationalization of dexamethasone dosage to mitigate this risk, particularly in the era of modern triple‐agent anti‐emetic regimens.

## Introduction

Adjuvant chemotherapy is associated with improved survival in early breast cancer (EBC) and is used as standard treatment for many patients following surgery. We present a case of *Pneumocystis jirovecii* pneumonia (PJP)—a rare and under‐recognized complication with potentially fatal consequences—in an otherwise well patient undergoing adjuvant chemotherapy in a dose‐dense fashion.

## Case Report

A 70‐year‐old Asian female underwent a wide local excision and sentinel lymph node biopsy for a Stage 1A (T1c N0 M0), grade two, triple‐negative invasive apocrine tumour of the breast with focal lymphovascular invasion and a Ki67 of 15% overall and 30% in hotspots.

Relevant comorbidities included well‐controlled, non‐insulin‐dependent, type two diabetes mellitus; hypertension; and dyslipidaemia. She was a lifelong non‐smoker.

She was commenced on adjuvant dose‐dense AC (ddAC) regimen 1, consisting of a single dose of Akynzeo® (fixed‐dose netupitant and palonosetron combination), dexamethasone 16 mg, doxorubicin 60 mg/m^2^, and cyclophosphamide 600 mg/m^2^ on Day 1, with pegfilgrastim on Day 2. Dexamethasone was continued at 8 mg on Days 2–3 and 4 mg on Days 4–5. The cycle was repeated every 14 days. She completed the first three cycles of treatment without incident. The day prior to her fourth cycle, she reported cough and low‐grade fever. She was commenced on a course of oral amoxicillin after a review by her general practitioner. Routine blood tests performed on that day were unremarkable, apart from lymphopenia (lymphocytes 0.26 × 10^9^/L, reference range 1.0–4.0 × 10^9^/L). She received her fourth cycle as scheduled. Eight days later, she reported severe fatigue, peripheral oedema, generalized weakness, and a cough and was admitted to hospital for investigation.

**Figure 1:**
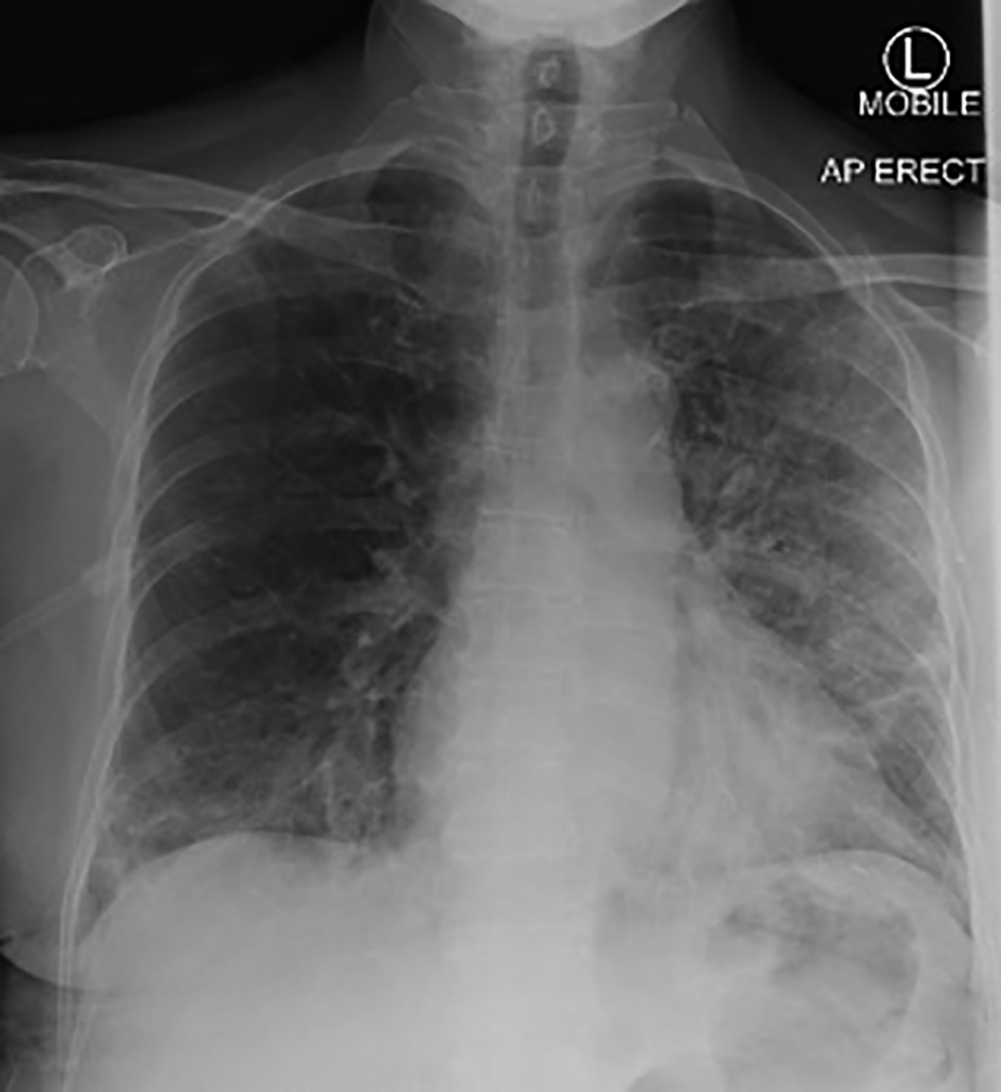
Chest X‐ray on admission demonstrated extensive patchy changes in both lungs but worse on the left.

On examination, she was hypoxic, with SpO_2_ by pulse oximetry of 88–90% on room air. Her other vital signs and physical examination were non‐contributory. Baseline bloodwork demonstrated a raised C‐reactive protein (CRP) of 151 mg/L (reference range, 0.0–5.0 mg/L) and lymphopenia (lymphocytes 0.31 × 10^9^/L; reference range, 1.0–4.0 × 10^9^/L). Chest X‐ray showed non‐specific patchy consolidation in the left lung and the right lung base (Fig. 1).

**Figure 2:**
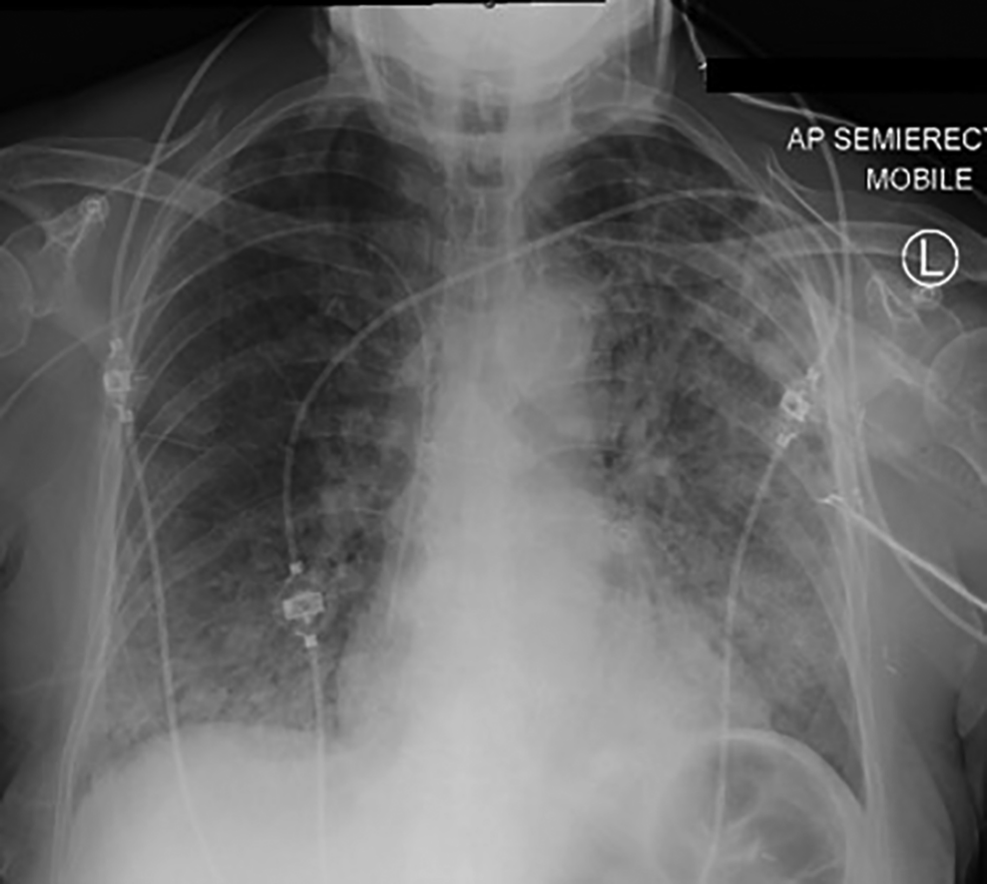
Chest X‐ray performed 36 h later demonstrated marked worsening of consolidation in both lungs.

Despite multiple antimicrobial agents for presumed community‐acquired pneumonia, her respiratory function continued to decline, with repeat chest X‐ray 36 h later showing extensive opacification in both lungs (Fig. 2). Over the subsequent days, she remained lymphopenic, with a nadir lymphocyte count of 0.29 × 10^9^/L. Simultaneously, progressive neutrophilia with left shift and rising CRP, consistent with toxic changes, were seen. On the fourth day of her admission, she was intubated for hypoxic respiratory failure. Until this point, extensive microbiology work‐up—including sputum and blood cultures, atypical serologies, respiratory virus polymerase chain reaction (PCR), and urinary Pneumococcal antigen—and an echocardiogram were all non‐contributory. Serum (1➔3)‐β‐D‐glucan was not available at our institution. A bronchoscopy was performed shortly after intubation, which was macroscopically normal. In consideration of the possibility of PJP, empirical high‐dose oral trimethoprim‐sulfamethoxazole was commenced after bronchoscopy.

Nine days following her bronchoscopy, *P. jirovecii* was detected by PCR on her bronchial washings. The long turnaround time for this result was because of a 4‐day laboratory closure over the Christmas holidays. Treatment was subsequently escalated to intravenous high‐dose trimethoprim‐sulfamethoxazole and methylprednisolone. Her condition continued to deteriorate, and she developed multi‐organ failure. Despite multiple life support measures, her condition failed to improve, and she eventually succumbed to the illness in the intensive care unit. Human immunodeficiency virus (HIV) testing was never performed in this patient as it was felt she did not have the risk factors for it.

## Discussion

This report describes a case of PJP in an otherwise well patient receiving adjuvant ddAC for EBC.

Since the early 2000s, dose‐dense chemotherapy has become an attractive treatment option for EBC in many centres, following the pivotal Cancer and Leukemia Group B 9741 trial 1, in which a statistically significant improvement in disease‐free survival and overall survival for the dose‐dense chemotherapy arm was demonstrated. This study was followed by other randomized controlled trials over the subsequent years to confirm the survival benefit with the dose‐dense approach 2, 3, 4, 5. Although the results from these trials were conflicting, a recent meta‐analysis by the Early Breast Cancer Trialists' Collaborative Group (EBCTCG) 6, which evaluated over 21,000 patients from 16 randomized trials, had found a statistically and clinically significant reduction in the risk of disease recurrence [relative risk (RR) 0.83, *P* = 0.00004] and breast cancer death within 10 years (RR 0.86, *P* = 0.004) with dose‐dense chemotherapy, compared to chemotherapy every 3 weeks. Although the toxicity data are yet to be fully described, these significant findings are likely to have a practice‐changing impact in favour of dose‐dense regimens.

PJP is an opportunistic infection that occurs in immunosuppressed patients, including those undergoing chemotherapy for a broad range of solid organ malignancies 7, and the use of corticosteroids in the chemotherapy regimen has been consistently reported as a major risk factor for this complication 8. Whilst it has been described in HIV‐negative patients with advanced solid tumours, it is not a well‐recognized complication of EBC treatment, with data largely confined to single case reports or small case series only. More recently, Waks et al. identified 19 cases of PJP amongst a series of 2057 patients receiving the ddAC‐containing regimen for EBC 9. All 19 cases were diagnosed after at least three doses of AC chemotherapy on a dose‐dense schedule, giving an overall incidence of 0.6% (95% confidence interval 0.3–1.0%). No PJP was identified in 1001 patients treated with standard, 3‐weekly AC. Amongst the 19 who developed PJP, the median steroid exposure was 16.4 mg prednisone equivalents/day for a median of 64 days. Of the 19 cases of PJP, one case was associated with a fatal outcome (5%), although historical data have consistently demonstrated a mortality risk of 30–60% amongst the HIV‐negative population, with cancer patients faring the worst 10. This study reinforces our knowledge that increased steroid dose and chemotherapy density is linked to an increased vulnerability to PJP. Despite this, there is a paucity of clinical efficacy data and consensus regarding the use of chemoprophylaxis in this vulnerable population group. In view of the small, but real, risk of complication of PJP in this group of patients who are likely to have excellent cancer‐specific survival, rationalization of the use of dexamethasone for patients receiving chemotherapy for EBC, particularly dose‐dense regimens, is warranted.

Corticosteroids have long been used in the prevention and treatment of chemotherapy‐induced nausea and vomiting (CINV). The introduction of modern anti‐emetic agents has transformed the landscape of CINV prevention, with many consensus guidelines (Table 1) now recommending a combination of a second‐generation 5‐HT_3_ receptor antagonist, an NK_1_ receptor antagonist, and dexamethasone on the day of chemotherapy administration. The recommendation for dexamethasone, however, is inconsistent across the various guidelines, with the American Society of Clinical Oncology (ASCO) 11 and the Multinational Association of Supportive Care in Cancer and European Society of Medical Oncology (MASCC‐ESMO) 12 guidelines recommending limiting dexamethasone to the day of chemotherapy only, whilst the National Comprehensive Cancer Network (NCCN) 13 guideline recommends a 3‐day course of dexamethasone. EviQ, the Australian point‐of‐care resource of evidence‐based, consensus‐driven cancer treatment protocols and information, recommends a 4‐day course of dexamethasone in combination with Akynzeo® on Day 1 for patients undergoing ddAC, although in June 2018, a footnote was added to allow the dosage and duration of dexamethasone from Days 2 to 4 to be reduced or omitted at physicians' discretion 14. Of note, the Phase III study comparing Akynzeo® with palonosetron in over 1400 women receiving AC utilized 12 mg of dexamethasone on Day 1 only 15. Further lending support to a dexamethasone‐sparing regimen was the randomized double‐blinded, placebo‐controlled, Phase III study by Ito et al. 16, which demonstrated the non‐inferiority of a dexamethasone‐sparing regimen over a 3‐day regimen in patients receiving highly emetogenic chemotherapy (HEC), when given with palonosetron and aprepitant.

**Table 1:** Current guidelines and recommendations for CINV prevention.

ASCO 10	In patients receiving the combination of an anthracycline and cyclophosphamide, dexamethasone can be limited to the day of chemotherapy administration. A four‐drug combination of an NK_1_ receptor antagonist, a 5‐HT_3_ receptor antagonist, dexamethasone, and olanzapine should be used, with olanzapine continued on Days 2–4
MASCC‐ESMO 11	In women with breast cancer, a three‐drug regimen, including single doses of a 5‐HT3 receptor antagonist, dexamethasone, and an NK_1_ receptor antagonist (aprepitant, fosaprepitant, netupitant or rolapitant), given before chemotherapy is recommended
	If fosaprepitant, netupitant, or rolapitant has been used on Day 1, no additional dexamethasone is required on subsequent days
NCCN 12	When used with netupitant/palonosetron, use 12 mg dexamethasone on Day 1 and 8 mg on Days 2 and 3, although “emerging data and clinical practice suggests dexamethasone dose may be individualized”
EviQ 13	Recommended supportive medication for ddAC:
	Akynzeo and 12 mg dexamethasone on Day 1
	8 mg dexamethasone on Days 2–4 (June 2018 update: may be reduced or omitted at physicians' discretion)

The patient in this case report received 40 mg of dexamethasone every 2 weeks as CINV prophylaxis or 19 mg prednisone equivalents/day. This higher steroid exposure is a reflection of older guidelines that predated the advent of novel anti‐emetic agents such as Akynzeo®.

In conclusion, PJP is a rare but potentially fatal complication of EBC treatment, especially in women on dose‐dense regimens. Increased awareness and vigilance amongst clinicians and measures to reduce the use of corticosteroid as supportive medication with chemotherapy are needed to mitigate this risk.

## Disclosure Statement

Appropriate written informed consent was obtained for publication of this case report and accompanying images.
